# Anti-Inflammatory Activity of Alkaloids: An Update from 2000 to 2010

**DOI:** 10.3390/molecules16108515

**Published:** 2011-10-11

**Authors:** Augusto Lopes Souto, Josean Fechine Tavares, Marcelo Sobral da Silva, Margareth de Fátima Formiga Melo Diniz, Petrônio Filgueiras de Athayde-Filho, José Maria Barbosa Filho

**Affiliations:** 1 Laboratory of Pharmaceutical Technology, Federal University of Paraiba, 58051-900, João Pessoa-PB, Brazil; Email: augustosouto@gmail.com (A.L.S.); josean@ltf.ufpb.br (J.F.T.); marcelosobral@ltf.ufpb.br (M.S.S.); margareth@ltf.ufpb.br (M.F.F.M.D.); 2 Department of Chemistry, Federal University of Paraíba, 58059-900, João Pessoa-PB, Brazil; Email: athayde-filho@quimica.ufpb.br

**Keywords:** alkaloids, anti-inflammatory activity, inflammation, experimental models, review

## Abstract

Many natural substances with proven anti-inflammatory activity have been isolated throughout the years. The aim of this review is to review naturally sourced alkaloids with anti-inflammatory effects reported from 2000 to 2010. The assays were conducted mostly *in vivo*, and carrageenan-induced pedal edema was the most used experimental model. Of the 49 alkaloids evaluated, 40 demonstrated anti-inflammatory activity. Of these the most studied type were the isoquinolines. This review was based on NAPRALERT data bank, Web of Science and Chemical Abstracts. In this review, 95 references are cited.

## 1. Introduction

Inflammation has been studied for thousands of years. Celsius (in 30 A.D.) described the four classical signs of inflammation (redness, heat, pain, and swelling), and used willow leaf extracts to relieve them [1]. 

The inflammatory process is a reaction of the body to the penetration of an infectious agent, an antigen, or cell damage. Inflammation is the most frequent sign of disease, and is also a fundamental biological process involving complex pathways that are often induced by the products of bacterial degradation from various microorganisms; lipopeptides, lipopolysaccharides, peptidoglycans, formylmethionyl peptides, flagellin, microbial DNA), fungi (zymosans), viruses (double-stranded RNA), or even the body’s own cells upon damage and death [2].

The inflammatory response starts with signal recognition that may have an infectious or inflammatory origin, and the release of chemicals from tissues and migrating cells called mediators [3]. The list of these mediators includes amines like histamine and 5-hydroxytryptamine, bradykinin, (representing short peptides), long peptides such as interleukin-1 (IL-1), lipids such as prostaglandins (PGs) and leukotrienes (LTs), and enzymes [1]. During the immune response, these mediators recruit adjacent cells through the paracrinal process. When these mediators exceed local borders, they disseminate, and distribute through the blood, producing endocrinal generalized cellular activation, or systematic inflammatory response syndrome (SIRS). SIRS is a host defense mechanism, and part of the tissue repair process. To effectively initiate this defense mechanism, cytokines with pro-inflammatory function are required, such as TNF-α, IL-1β, interleukin-12 (IL-12), interferon-γ (IFN-γ) and possibly IL-6 [4,5,6,7]. The initial inflammatory response is controlled by immune-regulating molecules through specific inhibitors, and soluble cytokine receptors. The main anti-inflammatory cytokines are transforming beta growth factor (TGF-β) and interleukins 4 and 10. Specific receptors for IL-1, TNF-α and interleukin-18 (IL-18) act as inhibitors of their own pro-inflammatory cytokines. Under physiological conditions immune-modulator molecules act to limit the potentially harmful effects of the inflammatory response [3]. The importance of each of these mediators can be seen when it is removed (either by preventing its generation with enzyme inhibitors or by preventing its pharmacological effects with selective antagonists) [1].

In inflammation research, several experimental models have been used to evaluate inflammation. The usual method of determining whether compounds have anti-inflammatory activity is to test them in animal, and biochemical inflammation models. However there is no single experimental model that covers all aspects of inflammation.

Natural products have long been recognized as an important source of therapeutically effective medicines. It is recognized that natural-product structures have great chemical diversity, biochemical specificity, and other molecular properties that make them favorable lead structures [8,9,10,11,12,13].

Among the 877 small-molecules New Chemical Entities (NCEs) introduced between 1981 and 2002, roughly 49% (~429 molecules) were natural products, semi-synthetic natural product analogues, or synthetic compounds based on natural-products [9], moreover, between 2005 and 2007, 13 natural, product-derived drugs were approved in the United States, with five of them being the first members of new classes [14]. In recent years advances in chemical and pharmacological techniques have contributed to the knowledge of new therapeutically active compounds obtained from natural products [15].

The alkaloids represent the largest single class of plant secondary metabolites. They have a remarkable range of often dramatic pharmacological activity, and are also often toxic to man [16]. Many alkaloids are used in therapeutics and as pharmacological tools. A wide range of biological effects has been reported for alkaloids, including emetic, anti-cholinergic, antitumor, diuretic, sympatho-mimetic, antiviral, antihypertensive, hypno-analgesic, antidepressant, mio-relaxant, anti-tussigen, antimicrobial and anti-inflammatory activities [17,18,19]. However, alkaloids and other natural compounds are generally complex, making it necessary to analyze their pharmacological activities using several experimental methods and demonstrate their structure/activity correlation. It is common to find pharmacological data where a single experimental model was used to demonstrate a biological activity. However pathological responses are extremely complex involving many biological events, so it is necessary to use different experimental models to define the exactly mechanism of action of the analyzed molecule [20].

In the course of our continuing search for bioactive natural plant products, we have published reviews on crude plant extracts and plant-derived compounds with potential uses [21,22,23,24,25,26,27,28,29,30,31,32,33,34,35,36,37]. Moreover, our group has also reviewed the medicinal and poisonous plants of Northeast Brazil [38,39], among others [40,41,42,43,44,45,46,47,48,49,50,51,52]. Recently we published a review on the anti-inflammatory activity of alkaloids reported in the twentieth century, more precisely covering the period from 1907 to 2000 [53]. Now we present an update of the literature on alkaloids with anti-inflammatory activity from 2000 to 2010. The search was carried out on data banks such as Web of Science, Chemical Abstracts, and NAPRALERT (acronym for the University of Illinois Natural Products ALERT service). The references found in the searches were later consulted. For details on the mechanism-based bioassays utilized for anti-inflammatory activity, the original references should be consulted.

## 2. Results and Discussion

Isoquinoline, quinoline and indole alkaloids were the most studied classes for anti-inflammatory activity. Among the isoquinolines, berberine was the most studied compound, being active in almost all the experimental models described in Table 1. This compound is present in numerous plants of the *Berberis* and *Coptis * genera [54]. It is one of the major components of *Coptis chinesis*, which is frequently utilized in Chinese herbal drugs to treat inflammatory reactions. Berberine has a variety of pharmacologic effects, including inhibition of TPA-induced mouse ear edema, indicating that this alkaloid may have activity against chronic inflammation [55].

Investigations demonstrated that warifteine, a bisbenzylisoquinoline alkaloid isolated from *Cissampelos sympodialis*, inhibits eosinophil recruitment, eotaxin and cisteinyl leukotriene production in the pleural cavities, and lungs of allergic mice, as well as inhibiting in the production of nitric oxide mediators. These data highlight the role of warifteine as a potential anti-allergic and anti-inflammatory molecule [56,57]. Other isoquinoline alkaloids like berbamine, palmatine and columbamine were also examined demonstrating significant dose-dependent inhibitory activity in serotonin-induced hind paw edema assays for both oral and topical applications, and in oral administration, on acetic acid-induced vascular permeability [58].

The quinolizidine alkaloids matrine and oxymatrine, isolated from *Sophora subprostrata* (a Chinese plant used as an antipyretic, antidote, and analgesic) exhibited *in vitro* cyclooxygenase inhibition and antioxidant activity, providing scientific support for their existing medicinal use in traditional Chinese medicine [59].

Indole alkaloids such as brucine and brucine-N-oxide were also reported in this review. They demonstrated significant analgesic and anti-inflammatory properties. Both compounds demonstrated a substantial protective effect in experimental models such as hot-plate test and writhing test. Although, in formalin test, they exhibited their analgesic activity in different phases. In carrageenan-induced rat paw edema experiment, brucine *N*-oxide showed stronger inhibitory effect than brucine. In addition, these two substances have diminished acetic-acid induced vascular permeability and inhibited the release of PGE_2_ in inflammatory tissue. These results suggest that brucine and brucine-*N*-oxide have different biochemical mechanisms, in spite of having similar chemical structure [60].

Marine natural products have been the focus for discovery of new chemical and pharmacological products. A bisindolic alkaloid named caulerpin isolated from the lipoid extract of the algae *Caulerpa racemosa* exhibited anti-inflammatory activity in mice when given orally at a concentration of 100 μmol/kg [63]. The bisindolic pharmacophoric nucleus of caulerpin is most likely responsible for the wide variety of biological properties tested; anti-inflammatory, antinociceptive [61] insecticidal [62], tumor inhibition [63], and inhibition of hypoxia transcription factor [64], all for this one alkaloid.

Amide alkaloids such as riparin I (*N*-benzoyl tyramine) and II (*N*-(2-hydroxybenzoyl) tyramine), isolated from the unripe fruit of *Aniba riparia* decreased carrageenan-induced paw edema at 4 h and 2 h respectively, when compared to a control [65,66]. It appears that the degree of hydroxylation of the benzoyl moiety increases the anti-inflammatory activity.

Most of the alkaloids reported in this review offer considerable promise as anti-inflammatory compounds or drug candidates and some of them appear to be remarkably active. The results of this search are presented in Table 1 in alphabetical order of their chemical names, followed by the plant species of origin. The references were consulted for details of the experimental models used while testing the alkaloid’s anti-inflammation activities (assay, organism tested, dose or concentration, activity, and references).

**Table 1:** Alkaloids with anti-inflammatory activity.

Substance and (Source)	Assay	Organism tested	Dose	Activity	Ref.
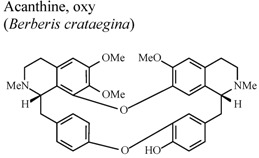	*In vivo*, 5-HT-Induced pedal edema	Mouse	200 mg/Kg	Inactive	[58]
	*In vivo*, 5-HT-Induced pedal edema	Mouse	200 mg/Kg	Active	[58]
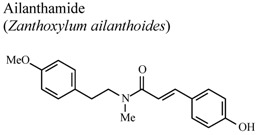	*In vivo*, inhibitory activity on superoxide generation by human neutrophils	Human	IC_50_ ≤ 5.34 µg/mL	Active	[67]
	*In vivo*, inhibitory activity on elastase release by human neutrophils	Human	IC_50_ ≤ 5.53 µg/mL	Active	[67]
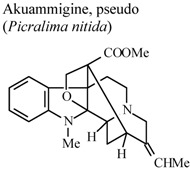	*In vivo*, carrageenan-induced pedal edema	Rat	1 mg/Kg	Active	[68]
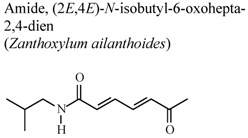	*In vivo*, inhibitory activity on superoxide generation by human neutrophils	Human	IC_50_ ≤ 5.34 µg/mL	Active	[67]
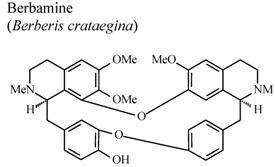	*In vivo*, 5-HT-induced pedal edema	Mouse	200 mg/Kg	Active	[58]
	*In vivo*, 5-HT-induced pedal edema	Mouse	100 mg/Kg	Active	[58]
	*In vivo*, TNB-induced colitis	Rat	15 mg/Kg	Active	[69]
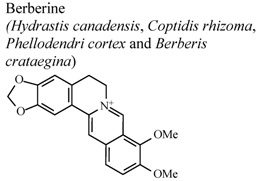	*In vivo*, LPS-induced hepatoxicity	Mouse	100 mg/Kg	Inactive	[70]
	*In vivo*, carrageenan-induced pedal edema	Mouse	2 mg/Kg	Active	[70]
	*In vivo*, LPS-induced hepatoxicity	Mouse	209 mg/Kg	Active	[70]
	*In vivo*, 5-HT induced-pedal edema	Mouse	200 mg/Kg	Active	[58]
	*In vivo*, Carrageenan-induced pedal edema	Rat	5 mg/Kg	Active	[55]
	*In vivo*, acute inflammation induced by *E. coli* LPS	Chicken	15 mg/Kg	Active	[71]
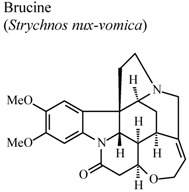	*In vivo*, carrageenan-induced pedal edema	Rat	15 mg/Kg	Active	[60]
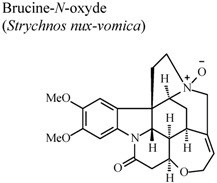	*In vivo*, carrageenan-induced pedal edema	Rat	100 mg/Kg	Active	[60]
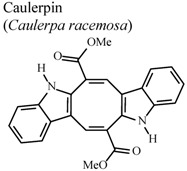	*In vivo*, capsaicin-induced ear edema	Mouse	100 µmol/Kg	Active	[61]
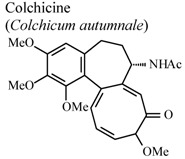	In humans, oral	Human adult	0.5 mg/person	Active	[72]
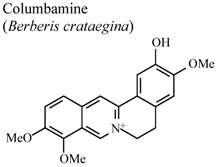	*In vivo*, External, 5-HT-induced pedal edema	Mouse	200 mg/Kg	Inactive	[58]
	*In vivo*, Intragastric, 5-HT-induced pedal edema	Mouse	200 mg/Kg	Inactive	[58]
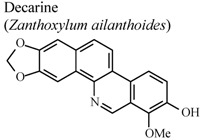	*In vivo*, inhibitory activity on superoxide generation by human neutrophils	Human	IC_50_ ≤ 5.34 µg/mL	Active	[67]
	*In vivo*, inhibitory activity on elastase release by human neutrophils	Human	IC_50_ ≤ 5.53 µg/mL	Active	[67]
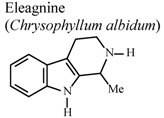	*In vivo*, carrageenan-induced paw edema	Rat	10 mg/Kg	Active	[73]
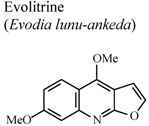	*In vivo*, carrageenan-induced rat paw edema	Rat	20 mg/Kg	Active	[74]
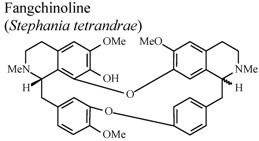	*In vivo*, croton oil-induced edema	Mouse	20 mg/Kg	Active	[75]
	*In vivo*, croton oil-induced edema	Mouse	0.1 mg/Kg	Active	[75]
	*In vitro*, fMLP-induced neutrophil adhesion and transmigration	Human	10 µg/mL	Active	[76]
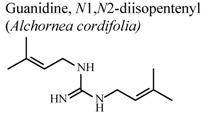	*In vivo*, croton oil-induced ear edema	Mouse	4.2 ± 0.5 mg/Kg	Active	[77]
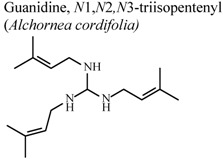	*In vivo*, croton oil-induced ear edema	Mouse	3.7 ± 0.8 mg/Kg	Active	[77]
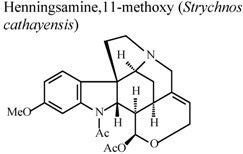	*In vitro*, inhibitory activity on superoxide anion generation	Human	IC_50_ < 5.5 5.43±1.52µg/mL	Active	[78]
	*In vitro*, inhibitory activity on elastase release by human neutrophils	Human	IC_50_ < 5.5 3.25 ± 0.31 µg/mL	Active	[78]
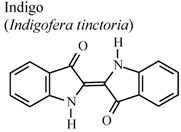	*In vivo*, carrageenan-induced pedal edema	Mouse	1 mg/Kg	Active	[79]
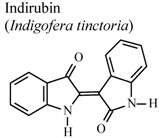	*In vivo*, carrageenan-induced pedal edema	Mouse	1 mg/Kg	Active	[79]
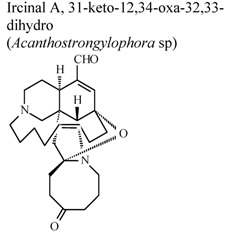	*	*	*	Inactive	[80]
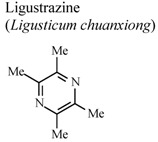	*In vitro*, macrophages	Human adult	400 mg/L	Active	[81]
	*In vivo*, carrageenan-induced pedal edema	Rat	50 mg/Kg	Active	[82]
	*In vivo*, Cotton pellet granuloma	Mouse	50 mg/Kg	Active	[82]
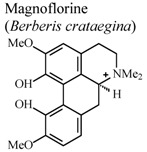	*In vivo*, 5-HT-induced pedal edema	Mouse	200 mg/Kg	Inactive	[58]
	*In vivo*, 5-HT-induced pedal edema	Mouse	200 mg/Kg	Inactive	[58]
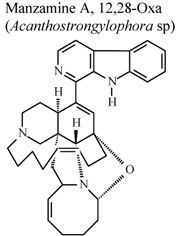	*	*	*	Inactive	[80]
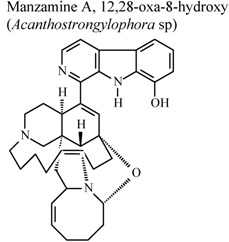	*	*	*	Inactive	[80]
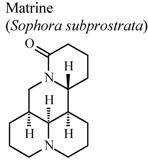	*In vitro*, inhibitory activity of COX-1	Rat	31.3 µM	Active	[59]
	*In vitro*, inhibitory activity of COX-2	Rat	188.5 µM	Moderate activity	[59]
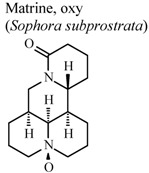	*In vitro*, inhibitory activity of COX-1	Rat	197.8 µM	Moderate activity	[59]
	*In vitro*, inhibitory activity of COX-2	Rat	385.1 µM	Weak activity	[59]
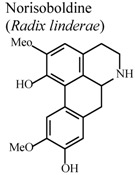	*In vivo*, collagen II -induced arthritis	Mouse	10 mg/Kg	Active	[83]
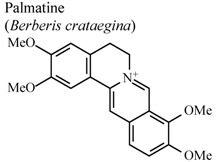	*In vivo*, 5-HT-induced pedal edema	Mouse	200 mg/Kg	Inactive	[58]
	*In vivo*, 5-HT-induced pedal edema	Mouse	100 mg/Kg	Active	[58]
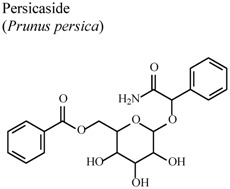	*In vitro*, inhibitory activity on NO production	Rat	40 µg/mL	Active	[84]
	*In vitro*, inhibitory activity on PGE_2_ production	Rat	40 µg/mL	Active	[84]
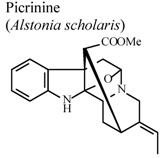	*In vitro*, inhibitory activity on COX-1	*	100 µM	Inactive	[85]
	*In vitro*, inhibitory activity on COX-2	*	100 µM	Weak activity	[85]
	*In vitro*, inhibitory activity on 5-LOX	*	100 µM	Active	[85]
	*In vivo*, carrageenan-induced air pouch formation	Mouse	10 mg/Kg	Active	[85]
	*In vivo*, xylene-induced ear edema	Mouse	10 mg/Kg	Active	[85]
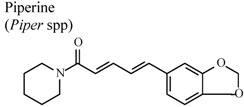	*In vivo*, intragastric	Rat	20 mg/Kg	Active	[86]
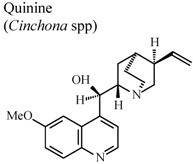	In humans, oral	Human adult	200 mg/day	Inactive	[87]
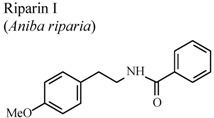	*In vivo*, formalin test	Mice	25 mg/Kg	Active	[88]
	*In vivo*, carrageenan-induced pedal edema	Mice	25 mg/Kg	Active	[65]
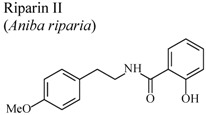	*In vivo*, carrageenan-induced pedal edema	Rat	25 mg/Kg	Active	[66]
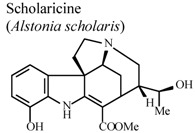	*In vitro*, inhibitory activity on COX-1	Mice	100 µM	Active	[85]
	*In vitro*, inhibitory activity on COX-2	Mice	100 µM	Active	[85]
	*In vitro*, inhibitory activity on 5-LOX	Mice	100 µM	Active	[85]
	*In vivo*, carrageenan-induced air pouch formation	Mouse	5 mg/Kg	Active	[85]
	*In vivo*, xylene-induced ear edema	Mouse	5 mg/Kg	Active	[85]
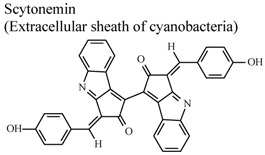	*In vitro*, phorbol-induced edema of the mouse ear	Mouse	5–100 µg/ear	Active	[89]
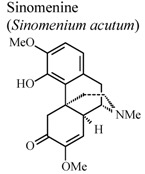	*In vivo*, collagen II induced arthritis	Rat	3.036 mg/Kg	Active	[90]
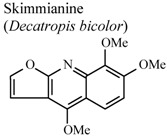	*In vivo*, TPA-induced inflammation	Mouse	0.75 mg/ear	Active	[91]
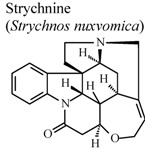	*In vivo*, carrageenan-induced pedal edema	Rat	*	Inactive	[92]
	*In vivo*, cotton pellet granuloma	Rat	*	Inactive	[92]
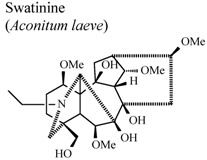	*In vitro*, colorimetric assay with tetrazolium salt	Blood drawn from healthy volunteers	100 µg/mL	Weak Activity	[93]
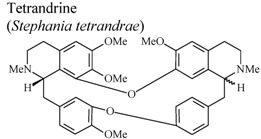	*In vivo*, croton oil-induced edema	Mouse	20 mg/Kg	Active	[75]
	*In vivo*, croton oil-induced edema	Mouse	0.1 mg/Kg	Active	[75]
	*In vitro*, FMLP-induced neutrophil adhesion and transmigration	Human	10 µg/mL	Active	[76]
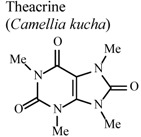	*In vivo*, xylene-induced ear edema	Mouse	8 mg/Kg	Active	[94]
	*In vivo*, acetic acid-induced vascular permeability	Mouse	16 mg/Kg	Active	[94]
	*In vivo*, carrageenan-induced paw edema	Mouse	8 mg/Kg	Active	[94]
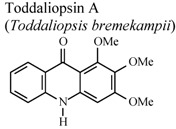	*In vitro*, zymosan activated human polymorphonuclear leucocytes in a chemoluminescence assay system	Human	IC_50_ = 27.3 µg/mL	Weak activity	[95]
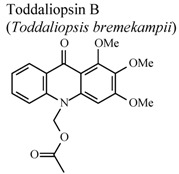	*In vitro*, zymosan activated human polymorphonuclear leucocytes in a chemoluminescence assay system	Human	IC_50_ = 48.3 µg/mL	Weak activity	[95]
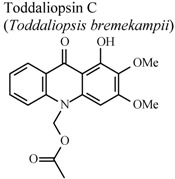	*In vitro*, zymosan activated human polymorphonuclear leucocytes in a chemoluminescence assay system	Human	IC_50_ = 4.21 µg/mL	Active	[95]
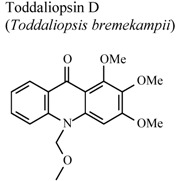	*In vitro*, zymosan activated human polymorphonuclear leucocytes in a chemoluminescence assay system	Human	IC_50_ = 79.1 µg/mL	Weak activity	[95]
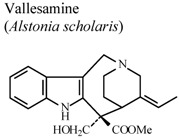	*In vitro*, inhibitory activity on COX-1	Mice	100 µM	Active	[85]
	*In vitro*, inhibitory activity on COX-2	Mice	100 µM	Active	[85]
	*In vitro*, inhibitory activity on 5-LOX	Mice	100 µM	Active	[85]
	*In vivo*, carrageenan-induced air pouch formation	Mouse	8 mg/Kg	Active	[85]
	*In vivo*, xylene-induced ear edema	Mouse	8 mg/Kg	Active	[85]
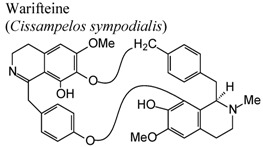	*In vivo*, allergic eosinophilia and cysteinyl leukotrienes production	Mice	50 μg/animal	Active	[56]
	*In vitro*. OVA-sensitized animals were evaluated. The response was related with the increase of NO production	Mice	0.4–10 mg/Kg	Active	[57]

* Data incomplete, derived from an abstract.

## 3. Conclusions

Of the 49 alkaloids evaluated, 40, among which the isoquinolines figured most prominently, demonstrated anti-inflammatory activity. Carrageenan-induced pedal edema was the most utilized experimental model for evaluating anti-inflammatory activity. In this review, 95 references were cited.

## References

[1] Vane J., Botting R. (1987). Inflammation and the mechanism of action of anti-inflammatory drugs. FASEB J..

[2] Kulinsky V.I. (2007). Biochemical aspects of inflammation. Biochemistry.

[3] Blancas-Flores G., Almanza-Pérez J.C., López-Roa R.I., Alarcón-Aguilar F.J., García-Macedo R., Cruz M. (2010). Obesity as an inflammatory process. Bol. Med. Hosp. Infant. Mex..

[4] Kim S., Choi M.G., Lee H.S., Lee S.K., Kim S.H., Kim W.W., Hur S.M., Kim J.H., Choe J.H., Nam S.J. (2009). Silibinin suppresses TNF-α-induced MMP-9 expression in gastric cancer cells through Inhibition of the MAPK pathway. Molecules.

[5] Lee D.C.W., Lau A.S.Y. (2011). Effects of *Panax ginseng* on tumor necrosis factor-α-mediated inflammation: a mini-review. Molecules.

[6] Chen L., He T., Han Y., Sheng J.Z., Jin S., Jin M.W. (2011). Pentamethylquercetin improves adiponectin expression in differentiated 3T3-L1 cells via a mechanism that implicates PPARγ together with TNF-α and IL-6. Molecules.

[7] Qiao Z., Ma J., Liu H. (2011). Effect of *Ligusticum wallichii* aqueous extract on oxidative injury and immunity activity in myocardial ischemic reperfusion rats. Int. J. Mol. Sci..

[8] Ekstein D., Schachter S.C. (2010). Natural products in epilepsy-the present situation and perspectives for the future. Pharmaceuticals.

[9] Koehn F.E., Carter G.T. (2005). The evolving role of natural products in drug discovery. Nature.

[10] McCloud T.G. (2010). High throughput extraction of plant, marine and fungal specimens for preservation of biologically active molecules. Molecules.

[11] Scotti L., Ferreira E.I., Silva M.S., Scotti M.T. (2010). Chemometric studies on natural products as potential inhibitors of the NADH. Molecules.

[12] Reis F.S., Pereira E., Barros L., Sousa M.J., Martins A., Ferreira I.C.F.R. (2011). Biomolecule profiles in inedible wild mushrooms with antioxidant value. Molecules.

[13] Kong K.W., Khoo H.E., Prasad K.N., Ismail A., Tan C.P., Rajab N.F. (2010). Revealing the power of the natural red pigment lycopene. Molecules.

[14] Li J.W.H., Vederas J.C. (2009). Drug discovery and natural products: End of an era or an endless frontier?. Science.

[15] Cechinel Filho V., Yunes R.A. (1998). Estratégias para obtenção de compostos farmacologicamente ativos a partir de plantas medicinais: Conceitos sobre modificação estrutural para otimização da atividade. Quim. Nova.

[16] Talib W.H., Mahasneh A.M. (2010). Antimicrobial, cytotoxicity and phytochemical screening of Jordanian plants used in traditional medicine. Molecules.

[17] Henriques A.T., Limberger R.P., Kerber V.A., Moreno P.R.H., Simões C.M.O., Schenkel E.P., Gosmann G., Mello J.C.P., Mentz L.A., Petrovick P.R. (2004). Alcalóides: Generalidades e Aspectos Básicos. Farmacognosia: Da Planta Ao Medicamento.

[18] Ezell S.J., Li H., Xu H., Zhang X., Gurpinar E., Zhang X., Rayburn E.R., Sommers C.I., Yang X., Velu S.E. (2010). Preclinical pharmacology of BA-TPQ, a novel synthetic iminoquinone anticancer agent. Mar. Drugs.

[19] Aiello A., Fattorusso E., Imperatore C., Irace C., Luciano P., Menna M., Santamaria R., Vitalone R. (2011). Zorrimidazolone, a bioactive alkaloid from the non-indigenous Mediterranean stolidobranch *Polyandrocarpa zorritensis*. Mar. Drugs.

[20] Falcão H.S., Leite J.A., Barbosa-Filho J.M., Athayde-Filho P.F., Chaves M.C.O., Moura M.D., Ferreira A.L., Almeida A.B.A., Souza-Brito A.R.M., Diniz M.F.F.M. (2008). Gastric and duodenal antiulcer activity of alkaloids: A review. Molecules.

[21] Moura M.D., Torres A.R., Oliveira R.A.G., Diniz M.F.F.M., Barbosa-Filho J.M. (2001). Natural products as inhibitors of models of mammary neoplasia. Br. J. Phytother..

[22] Moura M.D., Silva J.S., Oliveira R.A.G., Diniz M.F.F.M., Barbosa-Filho J.M. (2002). Natural products reported as potential inhibitors of uterine cervical neoplasia. Acta Farm. Bonaer..

[23] Silva J.S., Moura M.D., Oliveira R.A.G., Diniz M.F.F.M., Barbosa-Filho J.M. (2003). Natural product inhibitors of ovarian neoplasia. Phytomedicine.

[24] Gonçalves M.C.R., Moura L.S.A., Rabelo L.A., Barbosa-Filho J.M., Cruz H.M.M., Cruz J. (2000). Natural products inhibitors of HMG CoA reductase. Rev. Bras. Farm..

[25] Almeida R.N., Navarro D.S., Barbosa-Filho J.M. (2001). Plants with central analgesic activity. Phytomedicine.

[26] Pereira J.V., Modesto-Filho J., Agra M.F., Barbosa-Filho J.M. (2002). Plant and plant-derived compounds employed in prevention of the osteoporosis. Acta Farm. Bonaer..

[27] Morais L.C.S.L., Barbosa-Filho J.M., Almeida R.N. (2003). Plants and bioactive compounds for the treatment of Parkinson’s disease. Arq. Bras. Fitomed.Cient..

[28] Rocha L.G., Almeida J.R.G.S., Macedo R.O., Barbosa-Filho J.M. (2005). A review of natural products with antileishmanial activity. Phytomedicine.

[29] Barbosa-Filho J.M., Vasconcelos T.H.C., Alencar A.A., Batista L.M., Oliveira R.A.G., Guedes D.N., Falcão H.S., Moura M.D., Diniz M.F.F.M., Modesto-Filho J. (2005). Plants and their active constituents from South, Central, and North America with hypoglycemic activity. Rev. Bras. Farmacogn..

[30] Falcão H.S., Lima I.O., Santos V.L., Dantas H.F., Diniz M.F.F.M., Barbosa-Filho J.M., Batista L.M. (2005). Review of the plants with anti-inflammatory activity studied in Brazil. Rev. Bras. Farmacogn..

[31] Lima G.R.M., Montenegro C.A., Almeida C.L.F., Athayde-Filho P.F., Barbosa-Filho J.M., Batista L.M. (2011). Database survey of anti-inflammatory plants in South America: A review. Int. J. Mol. Sci..

[32] Barbosa-Filho J.M., Medeiros K.C.P., Diniz M.F.F.M., Batista L.M., Athayde-Filho P.F., Silva M.S., Cunha E.V.L., Almeida J.R.G.S., Quintans-Júnior L.J. (2006). Natural products inhibitors of the enzyme acetylcholinesterase. Rev. Bras. Farmacogn..

[33] Barbosa-Filho J.M., Martins V.K.M., Rabelo L.A., Moura M.D., Silva M.S., Cunha E.V.L., Souza M.F.V., Almeida R.N., Medeiros I.A. (2006). Natural products inhibitors of the angiotensin converting enzyme (ACE). A review between 1980-2000. Rev. Bras. Farmacogn..

[34] Amaral F.M.M., Ribeiro M.N.S., Barbosa-Filho J.M., Reis A.S., Nascimento F.R.F., Macedo R.O. (2006). Plants and chemical constituents with giardicidal activity. Rev. Bras. Farmacogn..

[35] Barbosa-Filho J.M., Nascimento-Júnior F.A., Tomaz A.C.A., Athayde-Filho P.F., Silva M.S., Cunha E.V.L. (2007). Natural products with antileprotic activity. Rev. Bras. Farmacogn..

[36] Falcão H.S., Mariath I.R., Diniz M.F.F.M., Batista L.M., Barbosa-Filho J.M. (2008). Plants of the American continent with antiulcer activity. Phytomedicine.

[37] Mota K.S.L., Dias G.E.N., Pinto M.E.F., Luiz-Ferreira A., Souza-Brito A.R.M., Hiruma-Lima C.A., Barbosa-Filho J.M., Batista L.M. (2009). Flavonoids with gastroprotective activity. Molecules.

[38] Agra M.F., França P.F., Barbosa-Filho J.M. (2007). Synopsis of the plants known as medicinal and poisonous in Northeast of Brazil. Rev. Bras Farmacogn..

[39] Agra M.F., Silva K.N., Basílio I.J.L.D., França P.F., Barbosa-Filho J.M. (2008). Survey of medicinal plants used in the region Northeast of Brazil. Rev. Bras Farmacogn..

[40] Silva F.L., Fischer D.C.H., Tavares J.F., Silva M.S., Athayde-Filho P.F., Barbosa-Filho J.M. (2011). Compilation of secondary metabolites from *Bidens pilosa* L. Molecules.

[41] Quintans-Júnior L.J., Almeida J.R.G.S., Lima J.T., Nunes X.P., Siqueira J.S., Oliveira L.E.G., Almeida R.N., Athayde-Filho P.F., Barbosa-Filho J.M. (2008). Plants with anticonvulsant properties-A review. Rev. Bras. Farmacogn..

[42] Sousa F.C.F., Melo C.T.V., Citó M.C.O., Félix F.H.C., Vasconcelos S.M.M., Fonteles M.M.F., Barbosa-Filho J.M., Viana G.S.B. (2008). Plantas medicinais e seus constituintes bioativos: Uma revisão da bioatividade e potenciais benefícios nos distúrbios da ansiedade em modelos animais. Rev. Bras. Farmacogn..

[43] Ribeiro-Filho J., Falcão H.S., Batista L.M., Barbosa-Filho J.M., Piuvezam M.R. (2010). Effects of plant extracts on HIV-1 protease. Curr. HIV Res..

[44] Barbosa-Filho J.M., Alencar A.A., Nunes X.P., Tomaz A.C.A., Sena-Filho J.G., Athayde-Filho P.F., Silva M.S., Souza M.F.V., Cunha E.V.L. (2008). Sources of alpha-, beta-, gamma-, delta- and epsilon-carotenes: A twentieth century review. Rev. Bras. Farmacogn..

[45] Alves J.S., Castro J.C., Freire M.O., Cunha E.V.L., Barbosa-Filho J.M., Silva M.S. (2000). Complete assignment of the ^1^H and ^13^C spectra of four triterpenes of the ursane, artane, lupane and friedelane groups. Magn. Reson. Chem..

[46] Sena-Filho J.G., Duringer J.M., Maia G.L.A., Tavares J.F., Xavier H.S., Silva M.S., Cunha E.V.L., Barbosa-Filho J.M. (2008). Ecdysteroids from *Vitex* species: Distribution and compilation of their ^13^C-NMR spectral data. Chem. Biodivers..

[47] Oliveira S.L., Silva M.S., Tavares J.F., Sena-Filho J.G., Lucena H.F.S., Romero M.A.V., Barbosa-Filho J.M. (2010). Tropane alkaloids from genus *Erythroxylum*: Distribution and compilation of ^13^C-NMR spectral data. Chem. Biodivers..

[48] Vasconcelos S.M.M., Honório-Júnior J.E.R., Abreu R.N.D.C., Silva M.C.C., Barbosa-Filho J.M., Lobato R.F.G., Varela A., Jasiah Ibañez J. (2009). Pharmacologic Study of Some Plant Species from the Brazilian Northeast: *Calotropis procera*, * Agava sisalana*, * Solanum paludosum*, * Dioscorea cayenensis* and *Crotalaria retusa*. Medicinal Plants: Classification, Biosynthesis and Pharmacology.

[49] Vasconcelos S.M.M., Pereira E.C., Chaves E.M.C., Lobato R.F.G., Barbosa-Filho J.M., Patrocínio M.C.A., Singh V.K., Govil J.N. (2010). Pharmacologic Study of *Amburana cearensis* and *Aniba* Genus. Recent Progress in Medicinal Plants. Drug Plant IV.

[50] Almeida C.L.F., Falcão H.S., Lima G.R.M., Montenegro C.A., Lira N.S., Athayde-Filho P.F., Rodrigues L.C., Souza M.F.V., Barbosa-Filho J.M., Batista L.M. (2011). Bioactivities from marine algae of the genus *Gracilaria*. Int. J. Mol. Sci..

[51] Barbosa-Filho J.M., Sette I.M.F., Cunha E.V.L., Guedes D.N., Silva M.S., Cordell G.A. (2005). Protoberberine Alkaloids. The Alkaloids.

[52] Conserva L.M., Pereira C.A.B., Barbosa-Filho J.M., Cordell G.A. (2005). Alkaloids of the Hernandiaceae: Occurrence and a Compilation of Their Biological Activities. The Alkaloids.

[53] Barbosa-Filho J.M., Piuvezam M.R., Moura M.D., Silva M.S., Lima K.V.B., da Cunha E.V.L., Fechine I.M., Takemura O.S. (2006). Anti-inflammatory activity of alkaloids: A twenty-century review. Rev. Bras. Farmacogn..

[54] Yoo K.Y., Hwang I.K., Kim J.D., Kang I.J., Park J., Yi J.S., Kim J.K., Bae Y.S., Won M.H. (2008). Anti-inflammatory effect of the ethanol extract of *Berberis koreana* in a gerbil model of cerebral ischemia/reperfusion. Phytother. Res..

[55] Kuo C.L., Chi C.W., Liu T.Y. (2004). The anti-inflammatory potential of berberine *in vitro* and *in vivo*. Cancer Lett..

[56] Bezerra-Santos C.R., Vieira-de-Abreu A., Barbosa-Filho J.M., Bandeira-Melo C., Piuvezam M.R., Bozza P.T. (2006). Anti-allergic properties of *Cissampelos sympodialis* and its isolated alkaloid warifteine. Int. Immunopharmacol..

[57] Costa H.F., Bezerra-Santos C.R., Barbosa Filho J.M., Martins M.A., Piuvezam M.R. (2008). Warifteine, a bisbenzylisoquinoline alkaloid, decreases immediate allergic and thermal hyperalgesic reactions in sensitized animals. Int. Immunopharmacol..

[58] Kupeli E., Kosar M., Yesilada E., Baser K.H.C, Baser C. (2002). A comparative study on the anti-inflammatory, antinociceptive and antipyretic effects of isoquinoline alkaloids from the roots of turkish berberis species. Life Sci..

[59] Ao C.W., Araki N., Tawata S. (2009). Cyclooxygenase inhibitory compounds with antioxidant activities from *Sophora subprostrata*. Asian J. Chem..

[60] Yin W., Wang T.S., Yin F.Z., Cai B.C. (2003). Analgesic and anti-inflammatory properties of brucine and brucine-*N*-oxide extracted from seeds of *Strychnos nuxvomica*. J. Ethnopharmacol..

[61] Souza E.T., Lira D.P., Queiroz A.C., Silva D.J.C., Aquino A.B., Mella E.A.C., Lorenzo V.P., Miranda G.E.C., Araújo-Júnior J.X., Chaves M.C.O. (2009). The antinociceptive and anti-inflammatory activities of caulerpin, a bisindole alkaloid isolated from seaweed of the genus *Caulerpa*. Mar. Drugs.

[62] Alarif W.M., Abou-Elnaga Z.S., Ayyad S.E.N., Al-Lihaibi S.S. (2010). Insecticidal metabolites from the green alga *Caulerpa racemosa*. Clean-Soil Air Water.

[63] Liu Y., Morgan J.B., Coothankandaswamy V., Liu R., Jekabson M.B., Mahdi F., Nagle D.G., Zhou Y.D. (2009). The *Caulerpa* pigment caulerpin inhibits HIF-1 activation and mitochondrial respiration. J. Nat. Prod..

[64] Ayyad S.E.N., Badria F.A. (1994). Caulerpine: An antitumor indole alkaloid from *Caulerpa racemosa*. Alex. J. Pharm. Sci..

[65] Leite C.P., Araújo F.L.O., Melo C.T.V., Gutierrez S.J.C., Barbosa-Filho J.M., Sousa F.C.F. (2011). Anti-inflammatory activity of riparin I (*O*-methyl-*N*-benzoyl tyramine) on paw edema models in mice. Inflamm. Res..

[66] Sousa F.C.F., Carvalho A.M.R., Leite C.P., Rocha N.F.M., Rios E.R.V., Vasconcelos L.F., Melo C.T.V., Lima S.T., Barbosa-Filho J.M., Vasconcelos S.M.M. (2011). Anti-inflammatory activity of riparin II (*N*-2-hydroxybenzoyl tyramine) in rats. Inflammat. Res..

[67] Chen J.J., Chung C.Y., Hwang T.L., Chen J.F. (2009). Amides and benzenoids from *Zanthoxylum ailanthoides* with inhibitory activity on superoxide generation and elastase release by neutrophils. J. Nat. Prod..

[68] Duwiejua M., Woode E., Obiri D.D. (2002). Pseudo-akuammigine, an alkaloid from *Picralima nitida* seeds, has anti-inflammatory and analgesic actions in rats. J. Ethnopharmacol..

[69] Zhou H., Mineshita S. (2000). The effect of berberine chloride on experimental colitis in rats *in vivo* and *in vitro*. J. Pharm. Exp. Ther..

[70] Yesilada E., Kupeli E. (2002). *Berberis crataegina* DC. root exhibits potent anti-inflammatory, analgesic and febrifuge effects in mice and rats. J. Ethnopharmacol..

[71] Shen Y.B., Piao X.S, Kim S.W., Wang L., Liu P. (2010). The effects of berberine on the magnitude of the acute inflammatory response induced by *Escherichia coli* lipopolysaccharide in broiler chickens. Poult. Sci..

[72] Das S.K., Ramakrishnan S., Mishra K., Srivastava R., Agarwal G.G., Singh R., Sircar A.R. (2002). A randomized controlled trial to evaluate the slow acting symptom-modifying effects of colchicine in osteoarthritis of the knee: A preliminary report. Arthritis Care Res..

[73] Idowu T.O., Iwalewa E.O., Aderogba M.A., Akinpelu B.A., Ogundaini A.O. (2006). Antinociceptive, anti-inflammatory and antioxidant activities of eleagnine: An alkaloid isolated from *Chrysophyllum albidum* seed cotyledons. J. Biol. Sci..

[74] Lal B., Bhise N.B., Gidwani R.M., Lakdawala A.D., Kalpana J., Patvardhan S. (2005). Isolation, synthesis and biological activity of evolitrine and analogs. ARKIVOC.

[75] Choi H.S., Kim H.S., Min K.R., Kim Y.S., Lim H.K., Chang Y.K., Chung M.W. (2000). Anti-inflamamtory effects of fangchinoline and tetrandrine. J. Ethnopharmacol..

[76] Shen Y.C., Chou C.J., Chiou W.F., Chen C.F. (2001). Anti-inflammatory effects of the partially purified extract of radix *Stephaniae tetrandrae*: Comparative studies of its active principles tetrandrine and fangchinoline on human polymorphonuclear leukocyte functions. Mol. Pharmacol..

[77] Manga H.M., Haddad M., Pieters L., Baccelli C., Penge A., Leclercq J.Q. (2008). Anti-inflammatory compounds from leaves and root bark of *Alchornea cordifolia* (Schumach. & Thonn.) Müll. Arg. J. Ethnopharmacol..

[78] Chen J.J., Luo Y.T., Hwang T.L., Sung P.J., Wang T.C., Chen I.S. (2008). A new indole alkaloid and anti-inflammatory constituents from *Strychnos cathayensis*. Chem. Biodivers..

[79] Ho Y.L., Tsai H.Y., Chang Y.S. (2001). Studies on the antinociceptive and antiinflammatory effects of indigo and indirubin in mice. J. Chin. Med. Sci..

[80] Yousaf M., Hammond N.L., Peng J.N., Wahyuono S., McIntosh K.A., Charman W.N.,  Mayer A.M.S., Hamann M.T. (2004). New manzamine alkaloids from an indo-pacific sponge. pharmacokinetics, oral availability, and the significant activity of several manzamines against HIV-I, AIDS opportunistic infections, and inflammatory diseases. J. Med. Chem..

[81] Peng Z., Zhang Z.X., Xu Y.J. (2000). Effect of ligustrazine on CD11c and CD14 content on alveolar macrophages from patients with chronic bronchitis. Zhongguo Yaolixue Yu Dulixue Zazhi.

[82] Fan Z., Liu Y. (2000). Anti-inflammatory effects of tetramethylpyrazine. Shenyang Yaoke Daxue Xuebao.

[83] Luo Y., Liu M., Xia Y., Dai Y., Chou G., Wang Z. (2010). Therapeutic effect of norisoboldine, an alkaloid isolated from *Radix Linderae*, on collagen-induced arthritis in mice. Phytomedicine.

[84] Rho J.R., Jun C.S., Ha Y.A., Yoo M.J., Cui M.X., Baek H.S., Lim J.A., Lee Y.H., Chai K.Y. (2007). Isolation and characterization of a new alkaloid from seed of *Prunus persica *L. and its anti-inflammatory activity. Bull. Korean Chem. Soc..

[85] Shang J.H., Cai X.H., Feng T., Zhao Y.L., Wang J.K., Zhang L.Y., Yan M., Luo X.D. (2010). Pharmacological evaluation of *Alstonia scholaris*: Anti-inflammatory and analgesic effects. J. Ethnopharmacol..

[86] Daware M.B., Mujumdar A.M., Ghaskabdi S. (2000). Reproductive toxicity of piperine in swiss albino mice. Planta Med..

[87] Williamson L., Illingworth H., Smith D., Mowat A. (2000). Oral quinine in ankylosing spondylitis: A randomized placebo controlled double blind crossover trial. J. Rheumatol..

[88] Araújo F.L.O., Melo C.T.V., Rocha N.F.M., Moura B.A., Leite C.P., Amaral J.F., Barbosa-Filho J.M., Gutierrez S.J.C., Vasconcelos S.M.M., Viana G.S.B. (2009). Antinociceptive effects of (*O*-methyl)-*N*-benzoyl tyramine (riparin I) from *Aniba riparia* (Nees) Mez (Lauraceae) in mice. Naunyn Schmiedebergs Arch. Pharmacol..

[89] Stevenson C.S., Capper E.A., Roshak A.K., Marquez B., Grace K., Gerwick W.H., Jacobs R.S., Marshall L.A. (2002). Scytonemin – a marine natural product inhibitor of kinases key in hyperproliferative inflammatory diseases. Inflamm. Res..

[90] Liu X.L., Chen G.X., Li X.J., Huang Q.C., Liu Q.P., Chen J.F. (2002). Inhibitive effects of sinomenine on inflammatory synovium in rats with arthritis induced by collagen II and its mechanism. Guangzhou Zhongyiyao Daxue Xuebao.

[91] Garcia-Argaez A.N., Ramirez Apan T.O., Delgado H.P., Velazquez G., Martinez-Varquez M. (2000). Anti-inflammatory activity of coumarins from *Decatropis bicolor* on TPA ear mice model. Planta Med..

[92] Xu L.J., Wei S.C., Lu F.E., Huang G., Huang G.Y., Ye W.Y. (2001). Comparison of effects of several components derived from *Strychnos nuxvomica* on experimental arthritis. Tongji Yike Daxue Xuebao.

[93] Shaheen F., Ahmad M., Khan T.H., Jalil S., Ejaz A., Sultankhodjaev M.N., Arfan M., Choudhary M.I., Rahman A.U. (2005). Alkaloids of *Aconitum laeve* and their anti-inflammatory, antioxidant and tyrosinase inhibition activities. Phytochemistry.

[94] Wang Y., Yang X., Zheng X., Li J., Ye C., Song X. (2010). Theacrine, a purine alkaloid with anti-inflammatory and analgesic activities. Fitoterapia.

[95] Naidoo D., Coombes P.H., Mulholland D.A., Crouch N.R., van Den Bergh A.J.J. (2005). *N*-Substituted acridone alkaloids from *Toddaliopsis bremekampii* (Rutaceae: Toddialoideae) of South-Central Africa. Phytochemistry.

